# Single food focus dietary guidance: lessons learned from an economic analysis of egg consumption

**DOI:** 10.1186/1478-7547-7-7

**Published:** 2009-04-14

**Authors:** Jordana K Schmier, Leila M Barraj, Nga L Tran

**Affiliations:** 1 Exponent, 1800 Diagonal Road, Suite 300, Alexandria, VA 22314, USA; 2 Exponent, 1150 Connecticut Avenue, NW, Suite 1100, Washington, DC 20036, USA

## Abstract

**Background:**

There is a large body of literature evaluating the impact of various nutrients of eggs and their dietary cholesterol content on health conditions. There is also literature on the costs of each condition associated with egg consumption. The goal of the present study is to synthesize what is known about the risks and benefits of eggs and the associated costs from a societal perspective.

**Methods:**

A risk apportionment model estimated the increased risk for coronary heart disease (CHD) attributable to egg cholesterol content, the decreased risk for other conditions (age-related macular degeneration (AMD), cataract, neural tube defects, and sarcopenia) associated with egg consumption, and a literature search identified the cost of illness of each condition. The base 795 case scenario calculated the costs or savings of each condition attributable to egg cholesterol or nutrient content.

**Results:**

Given the costs associated with CHD and the benefits associated with the other conditions, the most likely scenario associated with eating an egg a day is savings of $2.82 billion annually with uncertainty ranging from a net cost of $756 million to net savings up to $8.50 billion.

**Conclusion:**

This study evaluating the economic impact of egg consumption suggests that public health campaigns promoting limiting egg consumption as a means to reduce CHD risk would not be cost-effective from a societal perspective when other benefits are considered. Public health intervention that focuses on a single dietary constituent, and foods that are high in that constituent, may lead to unintended consequences of removing other beneficial constituents and the net effect may not be in its totality a desirable public health outcome. As newer data become available, the model should be updated.

## Introduction

High serum cholesterol levels are a major risk factor for cardiovascular disease, but unlike other major risk factors such as age, race, and gender, they can be modified to some extent. Only about one-fourth of low-density lipoproteins in the body are associated with diet and the remainder is produced by the liver or other cells in the body [1]. The two major strategies for managing and/or reducing cholesterol levels are a) pharmacological therapy, and b) therapeutic lifestyle changes. Pharmacologic therapies can include HMG CoA reductase inhibitors (commonly known as statins), selective cholesterol absorption inhibitors, renin inhibitors, fibrates, and niacin. These medications can also be used in combination. Therapeutic lifestyle changes include smoking cessation, increasing physical activity, and modifying one's diet, including keeping daily cholesterol intake less than 200 mg [2].

Dietary changes, while at first glance unequivocally positive, are not without possible detriments. Eliminating a source of cholesterol from the diet may create an opportunity for another, possibly more deleterious food. Also, foods containing cholesterol also contain other components, some of which may be beneficial. Balancing a reduction in cholesterol intake with complete nutritional needs is difficult and should consider maximizing other potential benefits.

One food that has been scrutinized in particular in terms of its nutritional value given its cholesterol content is the egg. Eggs are a good source of high quality protein as well as carotenoids such as lutein and zeaxanthin, and choline. Consumers of eggs are more likely to meet their recommended daily allowances of dietary folate and vitamins A, E, and B_12 _than non-egg consumers [3]. There is a large body of literature evaluating the impact of both these nutrients of eggs and their dietary cholesterol content on health conditions, including ophthalmic conditions, coronary heart disease, and neural tube defects, for example. There is also literature on the costs of each condition associated with egg consumption. The goal of the present study is to synthesize what is known about the risks and benefits of eggs and the associated costs. The basic framework is the findings of the Health Professionals' Follow-up Study (HPFS) and the Nurses' Health Study (NHS), which evaluated the health effects of egg consumption [4]. Using available data on the contribution of eggs to various conditions and existing estimates of the costs of each condition, this study estimates the economic impact of consumption of one additional egg daily.

## Methods

The analysis involved a multi-stage process. First, it was necessary to identify the conditions associated with egg consumption. Second, estimates of the contribution of egg consumption to each health risk or benefit were needed. Third, the economic impact of each of these conditions was required. Finally, the cost estimates were adjusted to reflect the contribution of eggs to each condition.

A literature search in PubMed was conducted to identify the conditions associated with the nutrients in eggs for which there was quantitative support. Based on the literature review, the proportion of each condition that would be influenced by the addition of one egg daily was estimated. A second PubMed search was conducted to identify publications estimating the costs of the conditions of interest. Abstracts were reviewed to identify publications with original data that assessed the cost of illness; reviews and comparative cost studies were excluded. The estimates were reviewed to determine the type of costs presented (e.g., direct medical vs. lost productivity), the population considered (e.g., Medicare-eligible vs. younger patients), and the year in which costs were estimated (in order to inflate to a common year).

For each condition, a "base case" was developed and a sensitivity analysis was conducted, assuming the minimum and maximum values as identified in the literature. If there were no minimum or maximum values to use for sensitivity analysis, the base case estimate was decreased and increased by 25% in order to arrive at extreme values. Similarly, if minimum and maximum values were available but not a specific base case, the midpoint of these extreme values was used as the base case input parameter.

## Results

The literature search identified a wide range of estimates for the costs of the conditions of interest, with various methods used to develop these estimates. A listing of the studies used to estimate costs and attribution in each area is provided in Table 1. Additional file 1 provides details on the methods for attribution.

**Table 1:** Relevant studies

**Condition**	**Studies Used to Estimate Cost (Base case, Min-Max)**	**Studies Used to Estimate Attribution (Percent Attributed, Min-Max)**
Coronary heart disease	Rosamond et al., 2007 [11] ($156.4B, $117B-$196B)	Barraj et al. 2008 [12] (0.30%, 0.10%–0.50%)
Eye health: Age-related macular degeneration	Halpern et al., 2006 [14]; Schmier et al., 2006a [18]; Schmier et al., 2006b [19]; Salm et al., 2006 [16], 2006; Rein et al., 2006 [15]; Coleman & Yu, 2008 [39] ($10.1B, $198.8 M-$19.96B)	Seddon et al., 1994 [35]; Handelman et al., 1999 [34]; Gale et al., 2003 [36]; Chung et al., 2004 [37] (9.5%, 4%–15%)
Eye health: Cataract	Rein et al., 2006 [15]; Schmier et al., 2007a (([21]; Schmier et al., 2007b [20] ($20.1B, $15.1B-$25.1B)	Handelman et al., 1999 [34]; Chasan-Taber et al., 1999 [32]; Vu et al., 2006 [33] (11%, 1%–21%)
Muscle mass: Sarcopenia	Janssen et al., 2004 [26] ($25.7 M, $16.1 M-$36.3 M)	Assumption (0.50%, 0.38%–0.63%)
Fetal health: Neural tube defects	Ouyang et al., 2007 [40]; Robbins et al., 2007 [41] ($52.0 M, $39.0 M-$64.9 M)	Shaw et al., 2004 [29]; Zeisel 2000 [38] (9%, 6.75%–11.25%)

### Coronary heart disease

While the role of the overall diet to coronary heart disease (CHD) risk is consistent, the evidence on dietary cholesterol is not always clear [5]. Recent epidemiological evidence has raised questions as to whether limiting dietary cholesterol intake would lead to any significant reduction in CHD risk [3,6-8]. Cholesterol feeding studies showed that increasing dietary cholesterol increases both low density lipoprotein (LDL) and high density lipoprotein (HDL) cholesterol with little change in the LDL:HDL ratio. This offers partial explanation for the lack of findings of an association between dietary cholesterol and coronary heart diseases [9]. Multiple other risk factors, including nonmodifiable ones such as age, gender, familial predisposition, and modifiable ones such as BMI and smoking, affect risk for CHD. One study suggested that blood cholesterol greater than 5.2 mmol/L may be associated with 43% of CHD in the United States in combination with other risk factors [10].

The American Heart Association estimates that the annual total direct and indirect costs of cardiovascular disease and stroke will be $448.5 billion in the United States in 2008 [11]. Both direct and indirect costs are included in this total, with direct costs including hospital, nursing home, physicians/other professionals, medical durables, and home health care, and indirect costs including lost productivity due to morbidity and mortality. CHD is responsible for $156.4 billion of this total. More than half (56%) of this is direct costs, while the remainder are indirect costs

The proportion of disease attributable to the intake of one egg daily is estimated at 0.3% based on recent evaluations [12]. The estimate is the average of estimates of 0.1% and 0.5% associated with the consumption of one egg per day (unpublished results, L. Barraj, N. Tran, P. Mink, D. McNamara) and two eggs per day [12]. The cost of CHD directly attributable to egg consumption was estimated at $469 million, with a range of $117 million to $978 million.

### Age-related macular degeneration

A recent study using data from the 2002 National Health Interview Survey estimated the prevalence of self-reported eye diseases in the United States among adults 18 and older [13]. Overall, the prevalence of AMD was 1.1%, suggesting that there are 331, 906 people with AMD in the US in 2007 [13].

Many studies have identified costs of AMD in the United States. These studies have used two primary approaches: claims data (generally from Medicare data) to identify direct medical costs or patient surveys to identify direct non-medical or indirect costs. Three studies are key to estimating direct ophthalmic costs [14-16]. Gupta and colleagues present a recent summary of what is known about the costs of AMD [17]. Halpern and colleagues [14] used Medicare data from 1999 to 2001 and calculated the total reimbursement per patient by AMD subtype. Rein and colleagues used MarketScan and Medicare data to estimate outpatient, inpatient, and prescription costs for AMD patients 40 years and older [15]. Another study comparing Medicare payments during two time periods also estimated the costs for the first year after diagnosis vs. follow-up costs [16]. Rehabilitation and counseling may be included in claims databases, but are more generally left out of cost analyses. Several studies have attempted to quantify non-medical costs. Few studies have identified non-medical costs associated with AMD in particular. The use of devices and the costs of these devices, for example, increased steadily as visual acuity decreased in patients with AMD [18]. Similarly, the use of caregiving increased as visual acuity decreased [19].

Based on the three studies that provided the comprehensive estimates of AMD-related costs, the minimum and maximum costs per case of AMD come to $599 to $60,135 (2007$) annually for medical costs, patient-reported services and devices, and caregiving. These estimates inflate the values from Halpern et al. [14] on direct medical costs from Medicare in 2001, with the minimum cost representing drusen only and the maximum cost representing wet AMD with photodynamic therapy. The second study includes the patient-reported costs for services and assistive devices associated with AMD with no visual impairment ($323) to vision of 20/250 or worse ($677) [19]. The third study reflects estimated costs for caregiving for patients with no visual impairment ($259) to vision of 20/250 or worse ($54,120) [18]. Finally, Rein and colleagues estimated that the cost was $415 for patients age 40 to 64 years and $627 for adults 65 and older. Although the prevalence of AMD is higher among older adults, to be conservative, this analysis took the mean of these two age group's costs.

After inflating costs to 2007 and multiplying these per-case annual costs by the estimated number of patients with AMD in the US, this analysis found that the reduction in expenditures associated with AMD resulting from adding an egg a day to the diet is $958 million, assuming a 9.5% reduction in AMD. The impact could range from $7.95 million (assuming per-patient costs of $599 with a 4% reduction in AMD) to $2.99 billion (assuming per-patient costs of $60,135 with a 15% reduction in AMD) (See Additional file 1.).

### Cataract

Cataract s a fairly common disease among the elderly, with a recent study reporting that 8.6% of all adults reported being diagnosed with cataract during their lifetime [13]. The lifetime prevalence increases dramatically with age, with 31% of adults age 65–74 and more than 53% of adults age 75 or older reporting having been diagnosed with cataract [13].

There is some information available about the cost of cataract in the United States [15], and also some data on the costs of common complications [20,21]. Rein and colleagues used data from MarketScan and Medicare to estimate the cost per patient based on outpatient, inpatient, and prescription medications for cataract. They estimated that the cost was $6957 for patients age 40 to 64 years and $6191 for adults 65 and older. The average of these two costs was used for this analysis.

Complications from cataract surgery can be very expensive. Two studies used Medicare data to estimate the incremental costs associated with cystoid macular edema (CME) and endophthalmitis after cataract surgery compared to patients who underwent cataract surgery and did not present with either complication. In 2005 dollars, ophthalmic costs were $1055 higher for patients who experienced CME and $3464 higher for patients with endophthalmitis [20,21]. Although the rates of these complications can vary, for the purpose of this estimate, it was assumed that CME occurred among 16% of patients (the midpoint of published estimates [22,23]) and endophthalmitis occurred among 0.1% [24].

The cost savings associated cataract with adding an egg a day to the diet were estimated at $2.21 billion, assuming a reduction in cataract rates of 11% (the midpoint between the low estimate of 1% and the high estimate of 21% (see Additional file 1) and per-patient annual costs of $7556 plus incremental costs for two common complications (CME and endophthalmitis, multiplied by the incidence of each complication).

### Skeletal muscle mass

Sarcopenia, a degenerative loss of skeletal muscle mass, is a common disease among older adults. Although difficult to diagnose, as neither definitive diagnostic criteria nor age-based thresholds exist, it is considered to be an important cause of disability among older adults [25]. Multiple causes may contribute to loss of muscle mass, and improved nutrition, including intake of high-quality animal protein, is just one of the possible methods to defer development of the condition and/or delay its progression.

One study was identified in the literature that estimated the cost of illness for sarcopenia. Janssen and colleagues [26] identified the costs of disability in the United States using standard national survey data and then calculated the fraction attributable to sarcopenia. They estimated that 1.5% of total health expenditures (a total of $18.5 billion in 2000) were associated with sarcopenia. Reducing sarcopenia by 10% would reduce costs by $1.1 billion, but this is dependent on whether that assumes patients with moderate disease avoiding the condition, or those with severe disease having a milder form [26].

While not enough is known about the epidemiology of sarcopenia to attribute the proportion of cases that might be prevented with the nutritional benefit of one egg daily with a high degree of precision, this analysis assumed, very conservatively, that 0.5% of cases of sarcopenia might be averted. Given the annual cost estimate of $25.7 billion (inflating the estimate to 2007), a reduction of one half percent of sarcopenia costs would result in $129 million savings annually in the United States. If the reduction were 25% less (an 0.45% reduction) or 25% greater (an 0.55% reduction), the savings would be $60 million to $227 million, accordingly.

### Neural tube defects

Many studies have evaluated the cost-benefit of folic acid fortification and increased intake in recent years. It has been suggested that if all women of child-bearing age were to adhere to the CDC recommendation of taking 400 mg of folic acid per day [27], approximately half of the annual cases of NTDs would be avoided. Grosse and colleagues compare and contrast existing studies and suggest that the annual economic benefit of folic acid fortification is between $312 to 425 million [28]. Based on Shaw et al [29], we estimated that that 9% of NTDs could be eliminated with the addition of one egg a day to the diet (see Additional file 1); to provide a range, this analysis assumed that the value could be 10% higher or lower. A recent study estimated health care expenditures of patients with spina bifida and found that total hospital charges for infants with spina bifida, anencephaly, and encephaloceles were $74.04 million, $1.1 million, and $10.9 million (respectively) in 2003. The other studies evaluated for this analysis included costs rather than charges. To account for the fact that hospital charges are greater than costs, an estimated cost-to-charge ratio was applied, with charges multiplied by 0.5 to estimate costs. Thus, after inflating costs to 2007 dollars, the estimated savings in healthcare expenditures associated with prevention of NTDs was $4.68 million, with a range from $2.63 to $7.31 million.

### Total costs associated with egg consumption

Based on these calculations, considering the costs associated with egg consumption associated with CHD and the benefits associated with AMD, cataract, neural tube defects and sarcopenia, the estimated annual savings are $2.82 billion annually (Table 2). Assuming the best case scenario, in which egg consumption has the least impact on CHD cost but the maximum impact on the other conditions evaluated, it is estimated that the savings associated with consumption of an egg per day could be as great as $8.39 billion. In the opposite scenario, in which egg consumption has the greater impact on CHD costs and the minimum impact on the other conditions evaluated, it is estimated that egg consumption could result in a net cost of $756 million annually.

**Table 2:** Estimate of costs and benefits from egg consumption

**Condition**	**Annual Costs/Benefits Associated with Egg Consumption** **Base case (Minimum, Maximum)**	**Comments**
CHD	$469 M ($117 M to $978 M)	Medical and nonmedical costs
AMD	-$958 M (-$7.95 M to -$2.99B)	Medical and nonmedical (caregiving, assistive devices) costs
Cataract	-$2.21B (-$151 M to -$5.28B)	Medical costs only, includes two common complications
Sarcopenia	-$129 M (-$60.4 M to -$227 M)	Medical costs only
NTD	-$4.68 M (-$2.63 M to -$7.31 M)	Hospital charges

## Discussion

The results presented in this study must be interpreted carefully. The two sources of data on which they are based, economic studies and data on the contribution of cholesterol to various conditions, are both subject to variation. However, each of the estimates provided here includes not only a base case value, but also a high and low value, based on the literature where possible, so that the full range of possibilities can be considered.

A number of limitations should be considered in terms of the cost estimates used in this analysis. While the cost estimates identified and used in this analysis were published, that does not imply that they are comprehensive. For example, no single economic study on any of the conditions of interest captured all the costs one might attribute. In most cases, direct medical costs are likely to be the primary source of disease-related costs, but lost productivity cannot be overlooked. In the case of AMD, a series of studies captured a variety of costs: direct medical [14], caregiving [19] and use of assistive devices and services [18]. The differences across studies make it difficult to compare literature-based values directly. Also, in the case of CHD, the annual cost estimates provided by the American Heart Association [11] address a wide variety of costs. The estimate for NTD, while well-constructed, is based on hospital costs [30]. The conversion from costs to charges reduces the precision of the estimate and the fact that the estimate is limited to hospitalizations is another limitation. It is also important to recognize that these factors mean that the estimates presented here are necessarily wide and still contain uncertainty. Future studies could replicate the format of this analysis, eventually narrowing the estimates. In the absence of such studies, this analysis represents a reasonable approximation of the economic impact of adding one egg daily to the diet.

This analysis also did not consider the cost of eggs. Compared with other protein sources, they are reasonably priced; we assumed zero additional cost for egg purchase. As with any other food, proper cooking is essential to limiting food-borne illness, particularly salmonella. The cooking process may increase the digestibility of eggs as well as reduce the possibility of illness. This model does not assume any additional cost associated with cooking eggs. Again, since other proteins that could be consumed in place of eggs would likely require cooking, this omission is appropriate.

It should be noted that the estimate of the fraction of CHD risk that can be attributed to eggs was based on an apportionment analysis that included a number of modifiable risk factors. One of the major considerations for applying the risk apportionment approach is the selection of appropriate risk factors and estimates of relative risks. The apportionment model included only multivariate-adjusted relative risk estimates derived from the same population, mainly from the HPFS and NHS. Both were chosen because they are large studies with long-follow-up periods and carefully collected information, and because the relative risk estimates were multivariate adjusted. It is possible that different estimates of the share of eggs to total CHD risk may have been derived if other assumptions were made about the potential interaction between the various risk factors, if other factors were included, or if risk estimates from other cohorts, e.g., population with different education levels or without the health background the participants in the HPFS and NHS had. Further the apportionment model used only considered the cholesterol effects of an egg, without considering other potentially beneficial components such as those described in this paper. Another important limitation to this analysis is the fact that considering any single food to the exclusion of others is inherently problematic. Kritchevsky and Kritchevsky [8] evaluated the studies estimating the relationship between egg consumption and coronary heart disease and found that very few had adjusted for other dietary intake (particularly total calories, fiber, and fat) and advise caution in interpreting these types of studies. Finally, it should be noted, that the published studies did not find any association between CHD risk and egg consumption, and there no statistically significant association between dietary cholesterol and CHD was found in the NHS and HPFS. The share of CHD attributed to eggs was modeled based on the cholesterol content of eggs and modeled association between dietary cholesterol, serum cholesterol and CHD [31-41]. This approach is likely to have resulted in an overestimate of the CHD risk estimate associated with egg consumption.

The implications of these findings for public health are two-fold. First, they echo those of several clinical studies that suggest that moderate egg consumption may not be harmful, and are indeed beneficial, among non-diabetic and non-hypercholesterolemic individuals. Well meaning public health campaigns designed to avoid eggs as a mean to lower serum LDL cholesterol and reducing CHD risk may be out of date and deserving of an update. If a cost-effectiveness study were conducted to evaluate public health campaigns limiting egg consumption as a means to reduce CHD risk, it may very well be discovered that these campaigns are not cost-effective from a societal perspective, particularly if the benefits of egg consumption are considered. Second, the human diet is a complex mixture of nutrients, antinutrients, functional components of varying degree of biological activities. Public health intervention that focuses on a single dietary constituent may lead to unintended consequences of removing other beneficial constituents and the net effect may not be a desirable public health outcome. The development of other examples would be a useful and interesting test of this hypothesis.

## Conclusion

These findings suggest that public health campaigns promoting limiting egg consumption as a means to reduce CHD risk are not cost-effective from a societal perspective when other benefits are considered. Public health interventions that focus on a single dietary constituent and foods that are high in that constituent may lead to unintended and undesirable consequences. As newer data become available, the model presented here should be updated. In particular, as there are conflicting data about CHD, including data suggesting that there may not be an increased risk, these findings may underestimate savings.

## Competing interests

The authors have no competing interests. All authors are employed at Exponent, Inc. Neither Exponent nor the authors have any financial ties or interests to the organization that provided funds to develop this manuscript, The Egg Nutrition Center, nor is the authors' employment dependent on the preparation or success of this project.

## Authors' contributions

NT and LB were involved in the initial conception of the project; JS helped refine the design. JS conducted the initial literature review and analysis; LB reviewed the calculations. JS drafted the manuscript; LB and NT provided critical review and comment. All authors read and approved the final manuscript.

## Supplementary Material

Additional file 1 **Quantifying egg benefits**. Technical appendix. Click here for file
