# Supplementary material for: Single food focus dietary guidance: lessons learned from an economic analysis of egg consumption
Source: Cost Eff Resour Alloc. 2009 Apr 14;7:7. doi: 10.1186/1478-7547-7-7 (PMC2672062; doi:10.1186/1478-7547-7-7)
Supplement: Additional file 1 — Quantifying egg benefits. Technical appendix. [file 1478-7547-7-7-S1.doc]

**Additional file 1. Quantifying egg benefits**

**Cataract**

The inverse dose-response relationship between the risk of cataract and Lutein + Zeaxanthin (L+Z) intake for females 45 years and older were derived from the NHS [32]. The quintiles of L+Z intakes and corresponding RR’s are summarized in Table A1. A more recent cross-sectional study conducted in adults 40 years or older in Melbourne [33] also provided quantifiable L+Z intake and corresponding RR’s, see Table A2. Based on NHS data and assuming a linear dose response model, a modest reduction in cataract risk (1%) were estimated from consuming an egg a day (an equivalent of approximately 505 μg Lutein + Zeaxanthin) [34]. The linear dose response derived from the Melbourne study [33] provides a larger estimate of cataract risk reduction (21%) from eating an egg a day, see Table A3.

Table A1. Dietary exposure to L+Z and risk of cataract, Nurses’ Health Study (Chasan-Taber et al. 1999 – Females >45y)

| **L+Z Intake Quintiles** | **Sample size** | **L+Z Intake µg/day** | **Multivariate RR*** | **95% CI** |
| --- | --- | --- | --- | --- |
| Quintile 1 | 295 | 1172 | 1 |  |
| Quintile 2 | 306 | 2064 | 1.01 | 0.86, 1.19 |
| Quintile 3 | 296 | 2817 | 0.95 | 0.8, 1.11 |
| Quintile 4 | 265 | 6047 | 0.81 | 0.69, 0.96 |
| Quintile 5 | 309 | 11685 | 0.88 | 0.75, 1.03 |
| Upper 10th percentile | 138 | 13701 | 0.78 | 0.63, 0.95 |
| *Adjusted for age, time period , diagnosis of diabetes (yes/no), cigarette smoking, BMI, areas of residence, no of physicians visits, aspirin use, total energy intake, and alcohol intake   - Linear Dose-Response Model: RR-1 = Slope x Dose - Slope: Risk Reduction per µg L+Z Intake = -1.43E-05 | | | | |

**Table A2. Dietary exposure to L+Z and risk of cataract (Vu et al. 2006, Adults >40 yrs)**

| **L+Z Intake Quintiles** | **Crude L+Z Intake (µg)** | **Crude L+Z Intake-Mid-Range (µg)** | **Multivariate Adjusted OR**** | **95%CI** |
| --- | --- | --- | --- | --- |
| Q1 | <454 | 454 | 1 |  |
| Q2 | 454-640 | 547 | 0.68 | 0.11-1.01 |
| Q3 | 640-812 | 726 | 0.62 | 0.40-0.96 |
| Q4 | 812-1104 | 958 | 0.59 | 0.38-0.93 |
| Q5 | >1104 | 1104 | 0.58 | 0.37-0.92 |
| **Adjusted for age, gender, iris color, diabetes, use of ACE inhibitor, use of beta blocker, use of loop diuretic, use of acetaminophen, myopia, AMD, and smoking   - Linear Dose Response Model: OR-1= Slope x Dose - Slope: Risk of Cataract Reduction per µg Intake = -4.13E-04 | | | | |

**Table A3. Egg’s L+Z and cataract risk reduction**

| **Reference** | **Slope Factor * Cataract Risk Reduction per µg L+Z Intake** | **Cataract Risk Reduction from an egg/day**** |
| --- | --- | --- |
| Chasan-Taber et al., 1999  Nurses Health Study  US Females >45 | -1.43E-05 | -1% |
| Vu et al., 2006  UK adults >45yrs | -4.13E-04 | -21% |

* Unweighted Linear Model: RR-1 = Slope * Dose

** An egg provides 505 μg L+Z

**AMD**

The dose response data for AMD risk and L+Z intake can be obtained from the large case control study by the Eye Disease Case-Control Study Group (EDCCS) [35]). The quintiles of L+Z intakes and corresponding adjusted odd ratios were extracted from Seddon et al. 1994 and summarized in Table A4. The dose-response relationship between plasma L+Z land AMD risks were available from Gale et al. [36] who investigated the relationship between plasma L+Z levels and AMD in a group of 380 men and women aged 66-75 from Sheffield, UK. The dose response data from this study is summarized in Table A5. Assuming a linear dose-response relationship and using the data from the EDCCS study [35], intake of one egg (providing 505 μg L+Z/day) was estimated to reduce risk of AMD by 4%, see Table A6. Converting dietary L+Z from one egg to plasma LZ levels using data from Handelman et al. ([34] and Chung et al. [37] and using the dose response data from Gale et al. [36] it was estimated that the minimum AMD risk reduction from consuming one egg a day 5 – 15%, see Table 6.

**Table A4. AMD and dietary lutein/zeaxanthin (Seddon et al. 1994: EDCCS)**

| **Quintiles** | **Median Lutein and Zeaxanthin Intake (IU noted in Table 2)** | **Lutein and Zeaxanthin intake (µg)** | **Multivariate Adjusted OR**** | **95%CI** |
| --- | --- | --- | --- | --- |
| Q1 | 560.8 | 560.8 | 1 |  |
| Q2 | 1211 | 1211 | 1.16 | 0.7-1.8 |
| Q3 | 1708 | 1708 | 0.85 | 0.5-1.4 |
| Q4 | 2487 | 2487 | 0.77 | 0.5-1.3 |
| Q5 | 5757 | 5757 | 0.44 | 0.2-0.8 |
| **Adjusted for age, sec, clinic, systolic BP, self reported physical activities, alcohol intake, BMI, smoking status, and Beta carotene in model   - Unweighted Model: RR-1 = Slope x Dose - Slope factor: AMD risk reduction per μg intake = -8.76E-05 | | | | |

Table A5. AMD and plasma L+Z (Gale et al., 2003)

| **Plasma level L+Z  (nmol/L)** | **Sample size** | **No. with AMD** | **Multivariate Adjusted OR**** | **95% CI** |
| --- | --- | --- | --- | --- |
| < 172.1 | 123 | 31 | 1.9 | 0.9-3.5 |
| 172.2-247.9 | 124 | 26 | 1.4 | 0.7-2.7 |
| >247.9 | 123 | 20 | 1 | 1 |
| **Adjusted for age, smoking, serum cholesterol, beer intake, history of angioplasty or coronary artery bypass, grafting, and hypermetropic refractive error.   - Unweighted Model: OR-1 = Slope x Dose - Slope Factor: AMD Risk Reduction per plasma Lutein and Zeaxanthin (nmol/L) = 8.51E-04 | | | | |

**Table A6. Egg’s intake (L+Z) and AMD risk reduction**

| Reference | Slope Factor * AMD Risk Reduction per unit L+Z Intake | Conversion mg L+Z intake to plasma level nmol/L*** | AMD Risk Reduction from one egg per day* |
| --- | --- | --- | --- |
| Seddon et al., 1994  EDCCS | -8.76E-05 (per μg) | -- | -4% |
| Gale et al., 2003 | 8.51E-04 (per nmol/L) | 230 | -10% |
| Gale et al., 2003 | 8.51E-04 (per nmol/L) | 110 | -5% |
| Gale et al., 2003 | 8.51E-04 (per nmol/L) | 350 | -15% |

* Unweighted Linear Model: RR-1 = Slope * Dose

** one egg provide 505 μg L+Z (Handelman et al., 1999)

***(Chung et al. 2004; Handelman et al., 1999)

**NTD**

Published data that could be used to quantify the potential health benefits from egg consumption are the inverse dose response relationship between maternal choline intake and NTD risk in Shaw et al. [29]. In this study, controlling for intake of supplemental folic acid, dietary folate, dietary methionine, and other covariates did not substantially influence risk estimates for choline. The dose response data from this study are summarized in Table A7. Based on these data and assuming a linear dose response mode, the reduction of NTD per each mg of choline intake is estimated to be -7.29E-04 (or 0.07% risk reduction per mg of choline intake). An egg provides an equivalent of 125 mg choline [38], hence consuming an egg a day could lead to as high as 9% reduction in NTD risk.

Table A7. Neural tube defects and preconception dietary intake of choline (Shaw et al., 2004)

| **Total Maternal Dietary Choline (mg/day)** | **n (cases)** | **n (controls)** | **Adjusted ORs **** | **95% CI** |
| --- | --- | --- | --- | --- |
| < 290.41 | 147 | 110 | 1 | -- |
| 290.42-371.52 | 99 | 110 | 0.63 | 0.42 - 0.99 |
| 371.53-498.46 | 98 | 110 | 0.65 | 0.39 - 1.07 |
| >498.46 | 80 | 110 | 0.51 | 0.25 - 1.07 |
| **Adjusted for pre-pregnant weight, height, education, race/ethnicity, preconception vitamin use, dietary folate, methionine, and energy intake in model   - Unweighted model: OR-1=slope x dose - Slope factor: NTD risk reduction per mg choline intake = -7.29 E-04 | | | | |
